# Recent Progress in a Membrane-Based Technique for Propylene/Propane Separation

**DOI:** 10.3390/membranes11050310

**Published:** 2021-04-23

**Authors:** Meng Guo, Masakoto Kanezashi

**Affiliations:** 1 Jiangsu Key Laboratory of Advanced Catalytic Materials and Technology, School of Petrochemical Engineering, Changzhou University, Changzhou 213164, China; guo@cczu.edu.cn; 2 Chemical Engineering Program, Graduate School of Advanced Science and Engineering, Hiroshima University, Higashi-Hiroshima 739-8527, Japan

**Keywords:** propylene/propane separation, polymeric membrane, inorganic membrane, hybrid membrane, organosilica membrane, pore size control, affinity control

## Abstract

The similar physico-chemical properties of propylene and propane molecules have made the separation process of propylene/propane challenging. Membrane separation techniques show substantial prospects in propylene/propane separation due to their low energy consumption and investment costs, and they have been proposed to replace or to be combined with the conventional cryogenic distillation process. Over the past decade, organosilica membranes have attracted considerable attention due to their significant features, such as their good molecular sieving properties and high hydrothermal stability. In the present review, holistic insight is provided to summarize the recent progress in propylene/propane separation using polymeric, inorganic, and hybrid membranes, and a particular inspection of organosilica membranes is conducted. The importance of the pore subnano-environment of organosilica membranes is highlighted, and future directions and perspectives for propylene/propane separation are also provided.

## 1. Introduction

Propylene (C_3_H_6_) plays a vital role in industrial applications because it is widely used for the production of downstream chemicals that are closely related to daily life [1,2]. For instance, most propylene is utilized to produce polypropylene and for the manufacture of commodities [1]. The annual production of C_3_H_6_ was approximately 100 million tons worldwide in 2016 and is expected to grow at a rate of 3.6% by 2025 [3]. To obtain high-purity C_3_H_6_, the separation of C_3_H_6_ from other components, such as propane (C_3_H_8_), is inevitable and is currently conducted by the cryogenic distillation process [1,2,4]. Nevertheless, the cryogenic distillation process consumes substantial energy due to the quite similar physical properties of C_3_H_6_ and C_3_H_8_ molecules (Table 1). Two huge splitter columns of 180 trays are usually used for the separation of C_3_H_6_/C_3_H_8_ in industry, which creates high capital and operating costs [5]. The intensive energy required for this process is estimated to equal the annual energy expenditure of Singapore [2]. In general, energy consumption is about 40% of total energy used in petrochemicals, estimated at about 1014 Btu per year. It also has an investment cost of over $50 million [6]. For this reason, even minor optimization of the purification process would have a significant impact on the energy savings for processing C_3_H_6_ and C_3_H_8_ mixtures [7]. Consequently, it is urgent to exploit new separation techniques with lower energy consumption.

**Figure 1:**
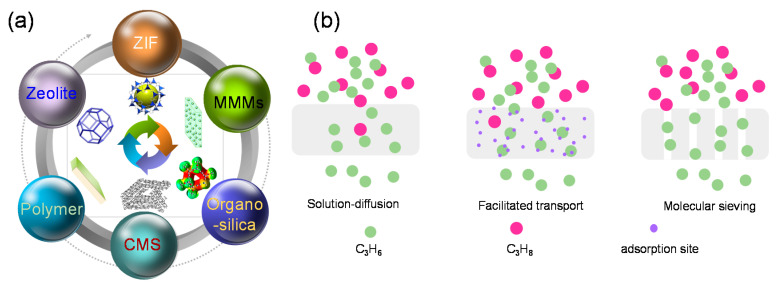
(**a**) Overview of the various membrane materials for C_3_H_6_/C_3_H_8_ separation. (**b**) General permeation mechanisms for C_3_H_6_/C_3_H_8_ separation through membranes.

Alternatively, separation processes based on absorption [8,9], adsorption [10,11], and membranes [12,13] have been extensively studied with the target of energy-efficient C_3_H_6_ purification. In comparison to the cryogenic distillation, these energy-saving techniques have shown much prospects in C_3_H_6_/C_3_H_8_ separation. Gas adsorption is a spontaneous process in which gas molecules adhere to the surface of the adsorbents. Indeed a lot of materials have been developed and displayed tremendous promise for propylene/propane separation, some issues still need to be addressed for further practical separation [7]. For instance, the high cost of the synthesized adsorbents and the regeneration issues after the adsorption. Membrane-based separation processes have attracted considerable attention due to their intrinsic advantages of lower energy use, continuous operation, and low investment costs [12,13,14,15]. In comparison to the energy-intensive cryogenic distillation, membrane based technology would use 90% less energy than distillation process [2]. A membrane with a C_3_H_6_ permeability of 1 barrer and C_3_H_6_/C_3_H_8_ selectivity of 35 was believed to have the high potential to compete with industrial cryogenic distillation [16,17]. However, it should be pointed out that permeance rather than the permeability is the direct indicator of real membrane-based separation performance, since the membrane thickness has no effect. In addition, a hybrid membrane-distillation configuration was proposed to improve the economics of C_3_H_6_/C_3_H_8_ separation. Koros and Lively clarified the target performance for a membrane to debottleneck the cryogenic distillation in a hybrid membrane-distillation configuration [18]. An economically acceptable process would require a membrane with a minimum C_3_H_6_ permeability of 10 barrer and C_3_H_6_/C_3_H_8_ selectivity of 20. Consequently, a variety of membrane materials have been developed, such as the polymeric membranes [19,20,21,22,23,24,25], mixed matrix membranes (MMMs) [26,27,28,29,30], carbon molecular sieve (CMS) membranes [31,32,33], zeolite membranes [34,35], and zeolitic imidazolate framework (ZIF) membranes, as illustrated in Figure 1a. However, the existence of intrinsic drawbacks still hampers the real application of these membranes for separating C_3_H_6_ and C_3_H_8_ mixtures. For instance, the large scale production of these membranes for C_3_H_6_/C_3_H_8_ separation is still difficult to be achieved. Organosilica membranes, an important part of the membrane family, have shown great applicability on various separation processes, including pervaporation, vapor permeation, reverse osmosis, and gas separations [36,37,38,39,40].

Generally, the mechanisms of C_3_H_6_/C_3_H_8_ separation through membranes can be classified into three categories, as presented in Figure 1b [13,41]. Polymeric membranes are dominated by a solution-diffusion model, where the adsorbed C_3_H_6_ and C_3_H_8_ molecules dissolve into the membrane materials and diffuse to the permeate side, driven by a concentration, pressure, or temperature gradient [42]. Another separation mechanism was attributed to the affinity between the membrane and the C_3_H_6_ molecules [43]. The specific affinity (formation of σ or π bonds) between the incorporated metal ions in the facilitated transport membranes and C_3_H_6_ molecules accelerated the preferential permeation of C_3_H_6_. Additionally, the permeation of C_3_H_6_ molecules through (organo)silica membranes can also be enhanced by the generation of hydrogen bonds between the silanol (Si-OH) groups and the π bonds in C_3_H_6_ [44,45]. The third separation mechanism is accepted as molecular sieving, which discriminates between C_3_H_6_ and C_3_H_8_ based on their molecular sizes or shapes. This mechanism is common in the CMS, ZIF-8, and organosilica membranes. Based on the discussion above, both the membrane’s molecular sieving properties and the affinities between C_3_H_6_ and the membrane matrix play decisive roles in the promotion of C_3_H_6_/C_3_H_8_ separation with an elevated efficiency.

In the past several decades, a wide variety of membranes have been extensively exploited, and many reviews have been presented that discuss C_3_H_6_/C_3_H_8_ separation. However, most of these reviews focused only on the polymeric membranes and ZIF-8 membranes. To the best of our knowledge, however, few specific papers have reviewed the development and the current status of C_3_H_6_/C_3_H_8_ separation through inorganic membranes, especially for the organosilica membranes. The fast development and the increased interest on the organosilica materials and membranes prompted us to summarize the current progress and consider the applications of C_3_H_6_/C_3_H_8_ separation more deeply. Holistic insight is first provided to summarize the propylene/propane separation using organosilica membranes. A brief summary of polymeric membranes and other types of inorganic membranes is also provided. Furthermore, the importance of the pore subnano-environment of organosilica membranes is highlighted, and future directions and perspectives for C_3_H_6_/C_3_H_8_ separation using organosilica membranes are presented.

## 2. Current Membrane Materials for C_3_H_6_/C_3_H_8_ Separation

### 2.1. Polymeric Membranes

The low costs and easy processability have widened the applications of polymeric membranes. Polyimide membranes, obtaining high mechanical properties, good thermal stability, and high chemical tolerance, are usually prepared from the polymerization reaction of dianhydride and diamine precursors [46]. However, the low permeability and plasticization effect at high pressure hamper the long-term operations [19,47]. Facilitated transport membranes were fabricated from the incorporation of metals with the target of overcoming the drawbacks of traditional polymeric membranes, such as low permeability and selectivity [5,48]. In comparison with the polymeric membranes, facilitated transport membranes display much potential for C_3_H_6_/C_3_H_8_ separation. Nevertheless, the further applications of these membranes were still restricted by the instability of the carriers.

#### 2.1.1. Polyimide Membranes

Polyimide (PI) membranes prepared from the polymerization reaction between di-anhydride and diamine precursors exhibit high chemical and mechanical stabilities. Thus, they have attracted considerable attention and are applied in C_3_H_6_/C_3_H_8_ separation [46]. A solution casting method was generally used for the fabrication of PI membranes. PI solutions were obtained by the polycondensation reaction using dianhydrides and diamines. Subsequently, the PI solution was casted onto the support and then vacuum drying process was further conducted. Finally, the supported PI membranes were fabricated. In Table 2, the C_3_H_6_/C_3_H_8_ separation performances of some representative polymeric membranes are listed. Nevertheless, the swelling and plasticization problems still perplex researchers and restrict further industrial scale-up. Koros’s group studied the permeation of C_3_H_6_/C_3_H_8_ mixtures through 4,4′-(hexafluoroisopropylidene)diphthalic anhydride (6FDA)-derived PI membranes [20]. The experimental results indicated that the separation performance is highly related to the types of precursors used during the membrane synthesis process. The fabricated PI membranes showed more permeability of C_3_H_6_ than C_3_H_8_, and the selectivity of C_3_H_6_/C_3_H_8_ ranged from 10–16. Nevertheless, the selectivity of C_3_H_6_/C_3_H_8_ mixtures was almost 50% lower than the ideal gas selectivity, even at low feed pressures. To solve the problem of plasticization in PI membranes, Das et al. proposed an approach to suppress the plasticization effect via a finely controlled annealing procedure for membrane fabrication [19]. No plasticization phenomenon was evident when the pressure was increased to 483 kPa at 70 °C. However, the low permeabilities of these polyimide membranes must still be improved.

Polymers of intrinsic microporosity (PIMs) comprising relatively inflexible macromolecular architectures with contortion sites have been shown to simultaneously boost the permeability and selectivity for membrane separation. Triptycene (KAUST-PI-1)- and spirobisindane (PIM-PI-1)-based PI membranes were systematically investigated for C_3_H_6_/C_3_H_8_ separation. The KAUST-PI-1 membrane displayed an ideal C_3_H_6_ permeability of 817 barrer with a C_3_H_6_/C_3_H_8_ selectivity of 16, allowing it to outperform the reported PI membranes [47]. The C_3_H_6_/C_3_H_8_ selectivity in the binary separation system, however, significantly dropped to 5 at a C_3_H_6_ partial pressure of 200 kPa because of the effect of plasticization and competitive sorption.

#### 2.1.2. Facilitated Transport Membranes

C_3_H_6_/C_3_H_8_ separation through polyimide membranes is dominated by the solution-diffusion mechanism. However, this mechanism is intrinsically not sufficient for distinguishing C_3_H_6_ and C_3_H_8_ molecules due to the similar physical properties of C_3_H_6_ and C_3_H_8_ molecules, as presented in Table 1 [13]. The small difference of the solubility between C_3_H_6_ and C_3_H_8_ molecules cannot result in effective discrimination. To promote the separation performance of C_3_H_6_/C_3_H_8_ mixtures, facilitated transport membranes have been exploited. The specific affinity between C_3_H_6_ molecules and metal ions cause the metal ions to act as the carriers, which facilitates the preferential sorption and diffusion of C_3_H_6_ through the membranes. As a result, both the C_3_H_6_ permeance and C_3_H_6_/C_3_H_8_ selectivity were obviously enhanced. Table 3 illustrates the C_3_H_6_/C_3_H_8_ separation performance of some facilitated transport membranes. The π-complexation intensity between the metals and the C_3_H_6_ molecules affect the carrier abilities of metals, which is determined by the electronegativity of the metal ion. Table 4 illustrates the electronegativities of some transition metals [5,48]. Generally, the higher electronegativity of a metal is, the stronger the intensity of the π-complexation becomes. Nevertheless, the excessive electronegativities of metals restrict the reversible reactivity and makes the desorption process of C_3_H_6_ more difficult. In contrast, metals with low electronegativities cannot provide a sufficiently high affinity for C_3_H_6_ molecules. The suitable electronegativity is reportedly in the range of 1.6–2.3, as studied by Kang et al. [48].

**Figure 2:**
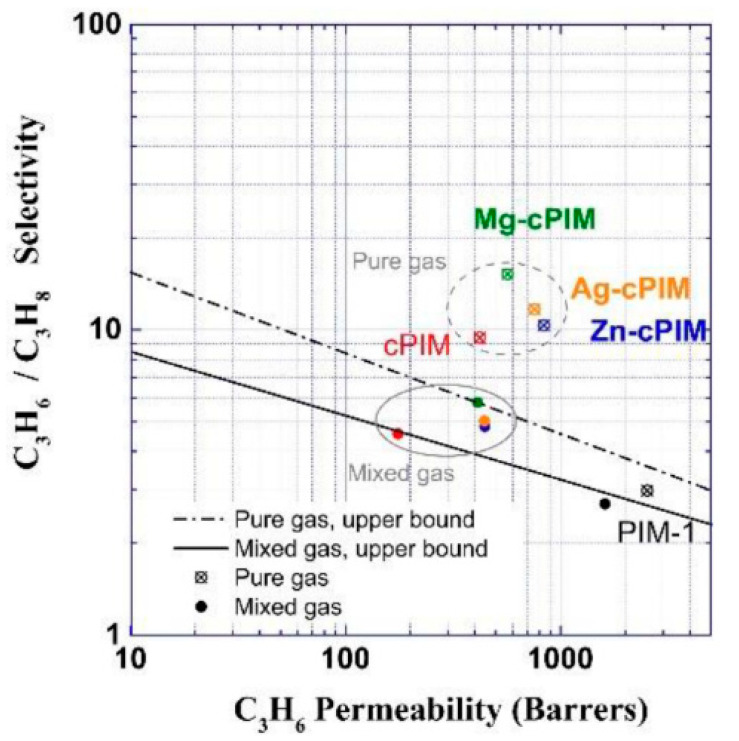
Trade-off of C_3_H_6_/C_3_H_8_ separation performance of pristine and metal-modified polymer of intrinsic microporosity (PIM) membranes [22].

Liao et al. explored C_3_H_6_/C_3_H_8_ separation behaviors by incorporating metal ions such as Zn^2+^, Mg^2+^, and Ag^+^ into PIMs [22]. Zn^2+^ increased the membrane permeabilities by enlarging the free volume of the matrix, while the affinity between the membranes and C_3_H_6_ molecules was enhanced after modification with Mg^2+^. The Ag^+^-functionalized PIMs resulted in a facilitated transport mechanism for the permeation of C_3_H_6_. In short, metal-ion-incorporated PIM membranes showed higher C_3_H_6_/C_3_H_8_ selectivities than those of pristine PIM membranes. The specific data for pristine and metal-modified PIM membranes for C_3_H_6_/C_3_H_8_ separation are shown in Figure 2. Kasahara et al. also reported facilitated transport in an ion-gel membrane with a high permeability of C_3_H_6_, which was attributed to the addition of silver ions [23]. However, the stability of facilitated transport membranes in C_3_H_6_/C_3_H_8_ separation requires further improvement. Jose et al. found that the C_3_H_6_/C_3_H_8_ selectivity of Ag-doped polymer membranes continuously decreased with the operation time due to the reduction of Ag^+^ during the reaction [24]. The agglomeration of Ag may lead to the appearance of defects, which is detrimental to the membrane separation performance.

### 2.2. Inorganic Membranes

In comparison to polymeric membranes, inorganic membranes composed of molecular sieves, such as CMSs, zeolites, and zeolite imidazolate frameworks (ZIFs), are important candidates for C_3_H_6_/C_3_H_8_ separation because of their high separation performances and excellent chemical and thermal stabilities. Table 5 briefly summarizes the characteristics and intrinsic drawbacks of these membranes.

#### 2.2.1. Carbon Molecular Sieve Membranes

**Figure 3:**
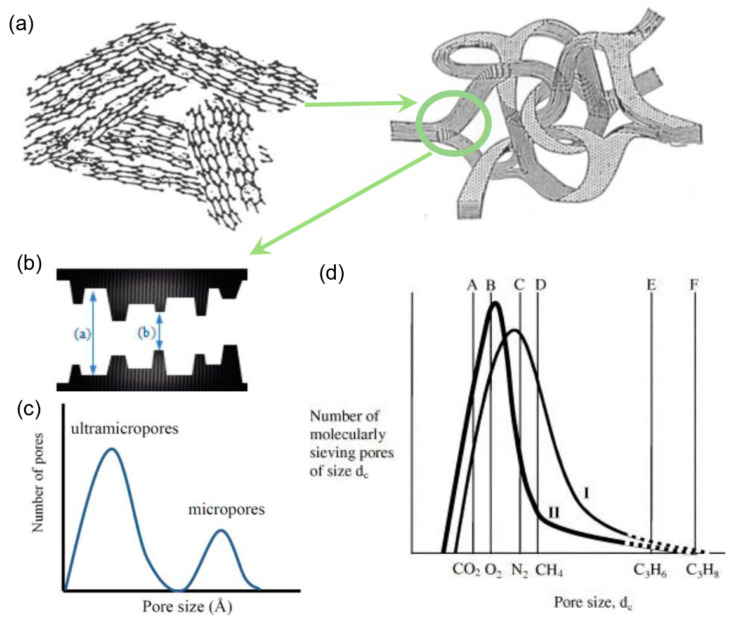
(**a**) Structure of pyrolytic carbon material [51]. (**b**) Schematic image of the “slit-like” structure and (**c**) the bimodal pore size distribution [49]. (**d**) Hypothetical semi-quantitative ultramicropore size distribution of CMS membrane [31].

CMS membranes fabricated from the pyrolysis treatment of polymers with intrinsic porosity yield an amorphous network structure and relatively broader distribution of the pore size [49]. The high processability also makes CMS membranes prepared via the pyrolysis of polymeric precursors suitable for C_3_H_6_/C_3_H_8_ separation. Steel and Koros examined the effect of the precursors and pyrolysis temperature on the microstructure and gas separation properties of CMS membranes [31]. The 6FDA/BPDA-DAM-derived membrane exhibited C_3_H_6_ permeability of 200 barrer and C_3_H_6_/C_3_H_8_ selectivity of 100 than Matrimid^®^-derived CMSs (permeability of 0.1 barrer and selectivity of 10, respectively) because of the larger fractional free-volume. In addition, the 6FDA-BPDA-DAM membrane pyrolyzed at 550 °C also exhibited much higher separation performances (permeability of 200 barrer and selectivity of 100) than that pyrolyzed at 800 °C (permeability of 1.3 barrer and selectivity of 7.9). A hypothetical model was proposed to explain this phenomenon, as shown in Figure 3. Both C_3_H_6_ and C_3_H_8_ could only permeate through the CMS membranes via the pores, as shown at the tail end of the hypothetical pore size distribution curve. However, the number of effective pores drastically decreased when the pyrolysis temperatures increased from 550 to 800 °C, and as a result, undesirable losses in permeability and selectivity were observed. Subsequently, Xu et al. found that the permeance decreased for the Matrimid^®^-derived CMS fiber membranes because of the increased thickness attributed to the structure collapse [32]. To suppress the collapse of the morphological structure for the CMS membrane, pre-pyrolysis treatment via a sol–gel crosslinking reaction was conducted [51]. A significant reduction in membrane thickness of up to 5–6 times (from ~16–17 to ~3–4 μm) was achieved, resulting in an efficient elevation in the membrane permeance.

The thickness can also affect the microstructure and separation properties of the CMS membrane [33]. The 6FDA-derived CMS membranes with thicknesses of 520 nm have exhibited C_3_H_6_/C_3_H_8_ permeance ratios of ~31 and C_3_H_6_ permeances of ~1.0 × 10^−8^ mol m^−2^ s^−1^ Pa^−1^ (~30 GPU, 1GPU = 3.348 × 10^−10^ mol m^−2^ s^−1^ Pa^−1^). As the thickness decreased to 300 nm, the permeance ratio of He/N_2_ increased, while that of C_3_H_6_/C_3_H_8_ decreased. The hypothetical qualitative model of the pore size distribution for CMS membranes proposed by Steel and Koros [31] was suitable for explaining this experimental phenomenon. The micropore size distribution was transformed to a region characterized by a smaller pore size as the membrane thickness decreased, which resulted in a significant reduction in the effective number of micropores available for C_3_H_6_ permeation. The scale-up of C_3_H_6_/C_3_H_8_ separation using CMS membranes, however, was difficult to achieve due to the low oxygen resistance and fragility. The specific performances of some CMS membranes are provided in Table 6.

#### 2.2.2. Zeolite Membranes

Zeolite membranes are drawing enormous interest due to their controllable pore sizes and strong adsorption properties. Different types of zeolite membranes have been extensively implemented for gas separations [34,35]. The faujasite (FAU)-type zeolite membranes with low Si/Al ratios were fabricated and applied for hydrocarbon separations for the first time by Nikolakis et al. [54]. The resultant membrane exhibited unsaturated hydrocarbon selectivity properties for benzene/cyclohexane, benzene/n-hexane, C_3_H_6_/C_3_H_8_, toluene/n-heptane, and ethylene/methane pairs. The size exclusion and the strong affinity between unsaturated hydrocarbons and zeolite cations are responsible for this. Giannakopoulos et al. also synthesized FAU membranes with thicknesses of 20 μm and examined the effect of operating temperatures and feed compositions for C_3_H_6_/C_3_H_8_ separation in detail [55]. A separation factor of 13.7 ± 1 was attained at 100 °C. Interestingly, the propane permeation was enhanced by the existence of propylene molecules in binary mixtures when the differences between the temperature-dependent single and binary C_3_H_6_/C_3_H_8_ separations were carefully analyzed.

**Figure 4:**
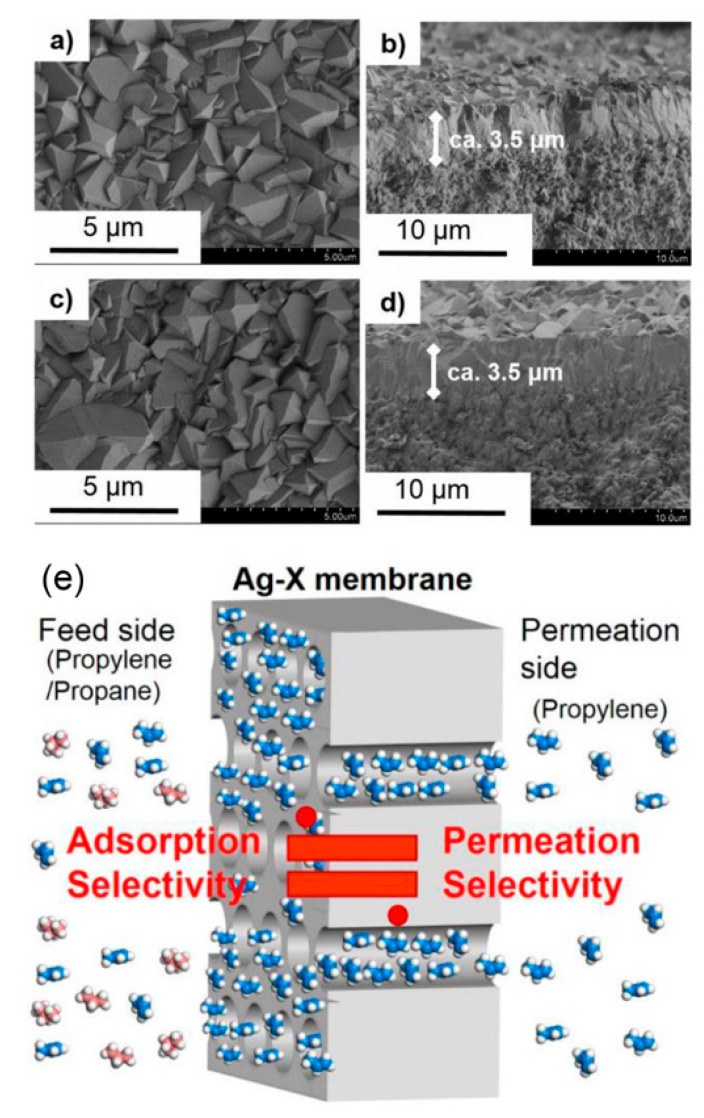
Typical field-emission scanning electron microscopy (FE-SEM) images of (**a**,**b**) Na-X and (**c**,**d**) Ag-X membrane ion exchanged with 10 mM AgNO_3_ solution [56]. (**e**) Schematic illustration of the relationship between the adsorption selectivity and permeation selectivity of the Ag-exchanged zeolite membrane [57].

Inspired by the facilitated transport mechanism, Sakai et al. reported an Ag-exchanged zeolite membrane that exhibited a high C_3_H_6_/C_3_H_8_ selectivity of 55 with a C_3_H_6_ permeance of 4.1 × 10^−8^ mol m^−2^ s^−1^ Pa^−1^ (122.5 GPU) at 80 °C, as shown in Figure 4 [56]. The C_3_H_6_/C_3_H_8_ selectivity was improved after the exchange of Na to Ag cations. The stability of Ag-exchanged zeolite membranes for C_3_H_6_/C_3_H_8_ separation was evidenced by the temperature-cycle measurements of binary gas separations. More importantly, no distinct decrease in membrane performance was measured after discontinuous use for more than one month. The authors claimed that the strong affinity between C_3_H_6_ and Ag cations was significant for the high separation performance. The adsorption properties of C_3_H_6_ and C_3_H_8_ on Ag-exchanged zeolite membranes confirmed the contribution of adsorption selectivity to the C_3_H_6_/C_3_H_8_ separation performance [57]. In view of the good consistency between the calculated C_3_H_6_ purity in the adsorbed phase and the C_3_H_6_ purity downstream of the Ag-exchanged zeolite membrane, the permeation of this membrane was estimated to be governed by the adsorption selectivity. However, non-selective defects are always formed in the zeolite membranes during the hydrothermal synthesis and high-temperature calcination process. The presence of these defects largely deteriorated the membrane separation performance. Consequently, the patching of defects in zeolite membranes has attracted much attention. Many strategies have been proposed to repair the defects, such as surface coating, chemical vapor deposition, coke deposition, rapid thermal processing, and hydrothermal treatment [58].

#### 2.2.3. Graphene Membranes

**Figure 5:**
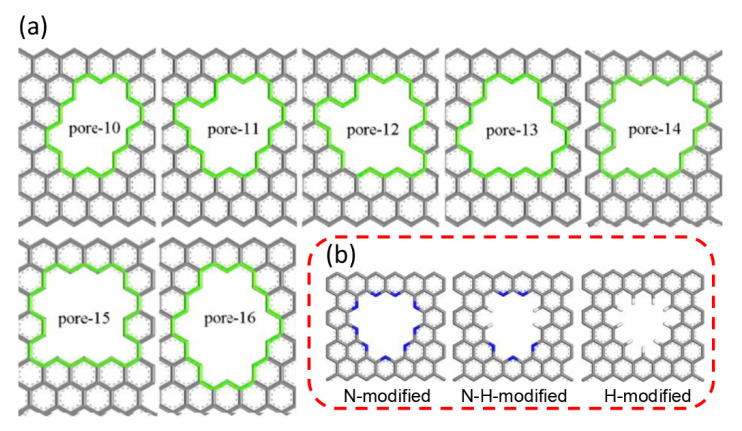
(**a**) Structure of porous graphene models. (**b**) Structure of the modified pore-13 [61].

Graphene, a 2D material consisting of sp^2^-hybridized carbon atoms, have been utilized for membrane-based gas separations after creating effective pores, since pristine graphene is inherently impermeable for any gases [59,60]. As one of the most promising materials, graphene, has attracted considerable attention. However, few practical graphene membranes have been reported for applications in C_3_H_6_/C_3_H_8_ separation. Nonetheless, some researchers have tried to predict the C_3_H_6_/C_3_H_8_ separation properties by utilizing molecular simulation approaches [61,62]. Jiang et al. conducted molecular dynamics and first-principles density functional theory to examine the separation behaviors of porous graphene membranes for C_3_H_6_ and C_3_H_8_ mixtures [61]. Molecular models of porous graphene membranes with different numbers of pores were attained by drilling carbon atoms on the original graphene, as shown in Figure 5a. In comparison with other membranes, the pore-13 membrane retained a good balance between the C_3_H_6_ permeability and C_3_H_6_/C_3_H_8_ selectivity. Nevertheless, the C_3_H_6_/C_3_H_8_ selectivity was still unsatisfactory. To further promote the separation performance, the decoration of the pore-13 membrane was implemented with N and H atoms, as shown in Figure 5b. The N-H-decorated membrane exhibited the best separation performance when compared to the N-modified and H-modified membranes. The N-H-decorated membrane possessed an attractive potential energy well for C_3_H_6_ and an energy barrier for C_3_H_8_, which enabled the easier permeation of C_3_H_6_ through the pores. Although the current experimental study of the utilization of practical porous graphene membranes for C_3_H_6_/C_3_H_8_ separation has progressed slowly, future work is still anticipated to be accelerated by the continuous development of advanced techniques.

#### 2.2.4. Zeolitic Imidazolate Framework (ZIF) Membranes

Metal organic framework (MOF) membranes have received a great deal of attention over the years due to their potential for use in high-efficiency gas and liquid purification [15,63,64]. Among the large family of MOF membranes, ZIF membranes, consisting of zinc ions bridged with the imidazolate ligands show great potential for hydrocarbon separations [63,65,66,67,68,69,70]. In this section, we mainly focus on the development of ZIF-8 and ZIF-67 membranes for the C_3_H_6_/C_3_H_8_ separation based on many reported studies. The specific separation performances of ZIF-8 and ZIF-67 membranes are listed in Table 7. Bux et al. first studied the gas permeation performances of ZIF-8 membranes in 2009, which exhibited outstanding H_2_/N_2_ and H_2_/CH_4_ separation properties [71]. The first report of C_3_H_6_/C_3_H_8_ separations utilizing ZIF-8 membranes was conducted by Pan et al. [65]. A defect-free ZIF-8 membrane fabricated by a hydrothermal seeded growth method showed superior performance for C_3_H_6_ and C_3_H_8_ mixtures. At the measurement temperature of −15 °C, a C_3_H_6_ permeance of 3 × 10^−8^ mol m^−2^ s^−1^ Pa^−1^ (90 GPU) and a C_3_H_6_/C_3_H_8_ selectivity of around 50 were surprisingly achieved. The first successful trial of C_3_H_6_/C_3_H_8_ separation by ZIF-8 membranes promoted subsequent extensive research work around the world.

**Figure 6:**
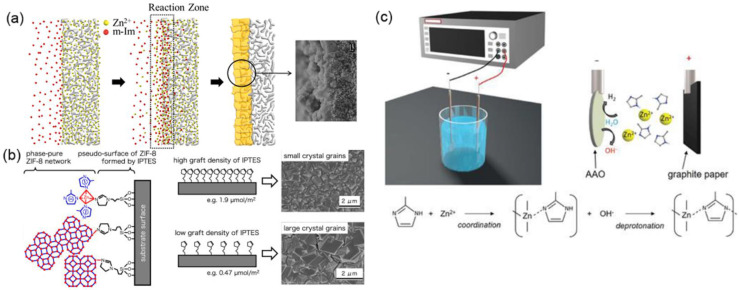
(**a**) Schematic illustration of membrane synthesis using the counter-diffusion method [17]. (**b**) Schematic image of the heterogeneous nucleation and crystal growth on the 3-(2-imidazolin-1-yl)propyltriethoxysilane (IPTES)-modified surface [73]. (**c**) Illustration of the experimental apparatus of atomic layer deposition (ALD) for the fabrication of ZIF-8 membranes [69].

In comparison with the hydrothermal seeded growth method, many other strategies have been exploited to fabricate ZIF-8 membranes for C_3_H_6_/C_3_H_8_ separation [15,63]. ZIF-8 membranes synthesized by a secondary growth method in aqueous solutions were evaluated based on the gas permeation, diffusion, and adsorption properties of C_3_H_6_ and C_3_H_8_ [72]. The secondary-growth-method-derived ZIF-8 membranes showed an over 30 times higher diffusivity for C_3_H_6_ (1.25 × 10^−8^ cm^2^ s^−1^) than for C_3_H_8_ (3.99 × 10^−10^ cm^2^ s^−1^). For C_3_H_6_ and C_3_H_8_ equimolar mixtures, ZIF-8 membranes exhibited stable separation performances (C_3_H_6_ permeance: 1.1 × 10^−8^ mol m^−2^ s^−1^ Pa^−1^, 33 GPU, C_3_H_6_/C_3_H_8_ selectivity: 30) during a discontinuous operation process for 40 days. A counter-diffusion concept was proposed by Kwon and Jeong for the in-situ synthesis of ZIF-8 membranes, as shown in Figure 6a [17]. The α-Al_2_O_3_ support was immersed in a metal ion solution and then transferred to a solvothermal growth environment. A reaction zone was formed by the diffusion of metal ions and ligands. Subsequently, a nucleation reaction occurred homogeneously in the vicinity of the support, resulting in the generation of high-quality ZIF-8 membranes. The resultant membrane showed a promising C_3_H_6_/C_3_H_8_ selectivity of 55.

Tanaka et al. reported a seeding-free aqueous synthesis strategy for the preparation of ZIF-8 membranes with controlled grain sizes [73]. The 3-(2-imidazolin-1-yl)propyltriethoxysilane (IPTES), acting as an imidazolate linker, was used to modify the surface of the membrane support. It was found that the grain sizes of the ZIF-8 membranes could be well controlled by fine-tuning of the grafting density of IPTES, as revealed in Figure 6b. The evaluation of single gas permeation measurement clearly indicated that the ideal C_3_H_6_/C_3_H_8_ selectivity increased with the IPTES density and approached 36, with a C_3_H_6_ permeance of 8.5 × 10^−8^ mol m^−2^ s^−1^ Pa^−1^ (254 GPU). Recently, an aqueously cathodic (ACD) deposition strategy was developed for the preparation of ZIF-8 membranes by Wei et al., as shown in Figure 6c [69]. No electrolyte, modulator, or organic solvent was added during the synthesis procedure, and thus, this is an eco-friendly and energy-saving approach. The resultant ZIF-8 membranes exhibited high C_3_H_6_/C_3_H_8_ separation performances (6.1 × 10^−8^ mol m^−2^ s^−1^ Pa^−1^ C_3_H_6_ permeance (182 GPU) and 142 C_3_H_6_/C_3_H_8_ selectivity), demonstrating great potential for the scalable application of ZIF-8 membranes.

**Figure 7:**
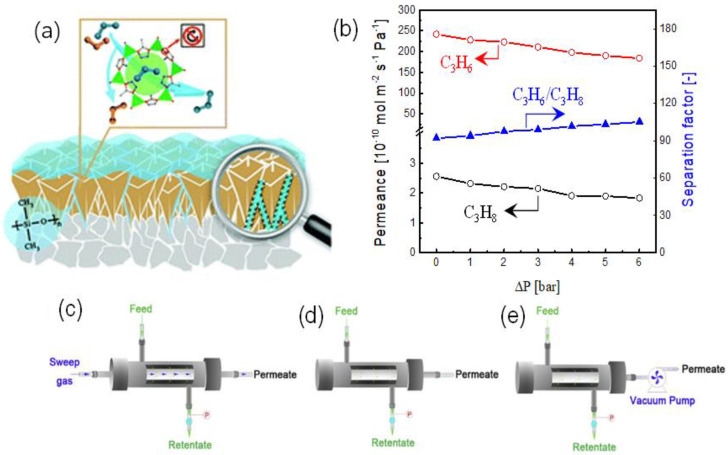
(**a**) Schematic illustration of the PDMS-coated ZIF-8 membrane [74]. (**b**) Separation properties of C_3_H_6_/C_3_H_8_ as a function of the transmembrane pressure [74]. Permeation conditions for C_3_H_6_/C_3_H_8_ separation (**c**) with sweep gas, (**d**) without sweep gas, and (**e**) with vacuum pump [75].

It is well known that the pressure-resistance properties and the defect issues are challenging for ZIF-8 membranes in C_3_H_6_/C_3_H_8_ separation [66]. Yu et al. reported the dramatic deterioration of the C_3_H_6_/C_3_H_8_ selectivity (from 61 to 14) as the transmembrane pressure drop increased (from 0 to 300 kPa), which may be attributed to the network flexibility rather than the permanent formation of defects because the separation performance could be easily recovered when the transmembrane pressure drop decreased. To solve this problem, Sheng et al. proposed a coating strategy using polydimethylsiloxane (PDMS) to simultaneously hinder the flexibility and patch the defects of ZIF-8 membranes, as shown in Figure 7a,b [74]. ZIF-8 membranes with PDMS coatings showed significantly enhanced C_3_H_6_/C_3_H_8_ selectivities and improved pressure resistance properties. The C_3_H_6_/C_3_H_8_ selectivity of hybrid PDMS-ZIF-8 membranes showed a slight increase from 93 to 105 when the transmembrane pressure increased from 0 to 600 kPa and could easily return to the initial level as the pressure reaches 0. In a subsequent study, Li et al. adopt a similar coating strategy to synthesize PDMS-ZIF-8 membranes on the porous tubular α-Al_2_O_3_ support for C_3_H_6_/C_3_H_8_ separation [75]. The effect of the operating pressure, temperature, and permeation conditions (with or without a sweep gas and a vacuum on the permeation side, as shown in Figure 7c–e, respectively) were carefully assessed. The permeation/separation behaviors of C_3_H_6_/C_3_H_8_ were found to be highly dependent on the permeation conditions. The conditions with a sweep gas and a vacuum pump were beneficial for the C_3_H_6_ permeation and the improved C_3_H_6_/C_3_H_8_ selectivity due to the quick removal of permeated C_3_H_6_. However, the operation with a vacuum pump was more desirable in practical applications than that using sweep gas, where the further separation of C_3_H_6_ and sweep gas is necessary.

**Figure 8:**
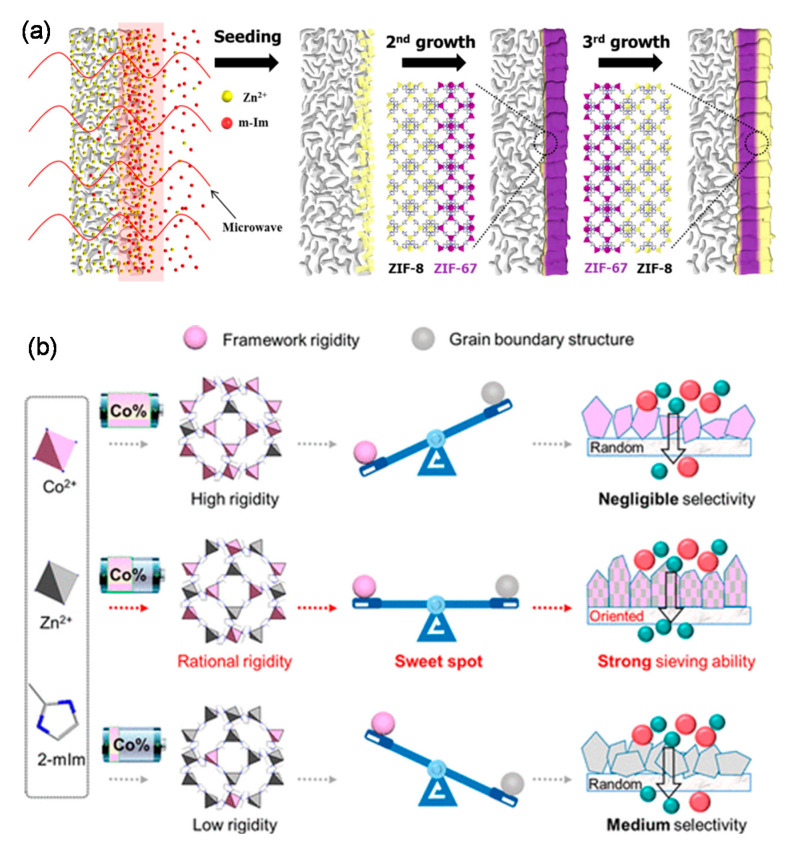
(**a**) Schematic illustration of the membrane synthesis via heteroepitaxial growth [76]. (**b**) Schematic illustration of the design of bimetallic Zn_(100-x)_Co_x_ZIF membranes [68].

In addition to the extensively studied ZIF-8 membranes, ZIF-67 membranes were also confirmed to be alternative candidates for C_3_H_6_/C_3_H_8_ separation [15,68,76]. Jeong et al. fabricated well inter-grown ZIF-67 membranes via heteroepitaxially growing ZIF-67 using ZIF-8 as the seed layers (Figure 8a), which exhibited an ultrahigh C_3_H_6_/C_3_H_8_ selectivity of approximately 200 along with a C_3_H_6_ permeance of 3.7 × 10^−8^ mol m^−2^ s^−1^ Pa^−1^ (106 GPU) [76]. In comparison with classical ZIF-8 membranes, the network structures of the ZIF-67 membranes were more rigid because the shorter Co-N bonds substituted the Zn-N bonds in the ZIF-8. However, few papers focused on using ZIF-67 membranes for C_3_H_6_/C_3_H_8_ separation with high selectivity. The low separation performance was possibly attributed to the poor grain boundary structure derived from the ultrafast crystallization reaction. In a recently published paper, Hou et al. proposed a crystal engineering strategy to balance the grain boundary structure and the flexibility within ZIF-67 membranes [68]. The Zn^2+^ was incorporated into the framework of ZIF-67 membranes to fabricate a series of bimetallic ZIFs membranes with different Co^2+^/Zn^2+^ ratios, as revealed in Figure 8b. A high C_3_H_6_/C_3_H_8_ separation performance could not be obtained in ZIFs membranes with Co^2+^/Zn^2+^ ratios that were too high or too low. The optimal balance between the grain boundary structure and the flexibility was attained by the membrane with a Co^2+^/Zn^2+^ ratio of 18/82, which exhibited a C_3_H_6_/C_3_H_8_ selectivity of around 175 with a C_3_H_6_ permeance of 2 × 10^−8^ mol m^−2^ s^−1^ Pa^−1^ (60 GPU).

### 2.3. Mixed Matrix Membranes (MMMs)

To combine the advantages of a superior porosity, high permeance of inorganic membranes, easy processability, and low cost of polymeric membranes, MMMs were intensively implemented for the target molecular separation [26]. Liu et al. reported the “conformation-controlled molecular sieving effect” on the C_3_H_6_/C_3_H_8_ separation of MMMs [27]. The incorporated Zr-fum-fcu-MOF in the 6FDA-DAM matrix, which had a contracted triangular pore-aperture, efficiently improved the C_3_H_6_/C_3_H_8_ separation performance and overcame the problem of the plasticization effect. The separation properties (C_3_H_6_ permeance: 20–30 barrer, C_3_H_6_/C_3_H_8_ selectivity: 15–20) far exceeded the upper bound of polymer membranes. Zhang et al. fabricated ZIF-8/6FDA-DAM membranes on the hollow fiber supports for possible scale-up applications [28]. The as-synthesized MMMs with a 30 wt% ZIF-8 loading exhibited a high C_3_H_6_/C_3_H_8_ selectivity of around 27.5. However, the C_3_H_6_ permeance was much lower than those of pure ZIF-8 membranes. Furthermore, the maximum loading of inorganic fillers of the aforementioned MMMs was limited to 30 wt% due to compatibility issues. Ma et al. reported that a ZIF-8 incorporated PIM-6FDA-OH membrane with a high loading ratio up to 65 wt% demonstrated a satisfying C_3_H_6_/C_3_H_8_ selectivity of 28.7 together a C_3_H_6_ permeability of 30 barrer and good plasticization resistance at a feed pressure of 700 kPa [29]. The improved compatibility was attributed to the (N···O-H)-induced hydrogen bonding between the ZIF-8 filler and the PIM-6FDA-OH matrix, as confirmed by Fourier-transform infrared spectroscopy (FT-IR) and X-ray photoelectron spectroscopy (XPS) characterization.

The reported MMMs exhibited higher C_3_H_6_ permeances and C_3_H_6_/C_3_H_8_ selectivities than those of pure polymer membranes, yet the performances were inherently restrained by the bulk polymer matrix. To exceed this limitation, Rashidi et al. first proposed the concept of “all-nanoporous hybrid membranes” based on ZIF-8 and MFI, in which both the dispersed and continuous phases were nanoporous materials [30]. The separation performance of the all-nanoporous ZIF-8/MFI membrane exceeded those of the individual ZIF-8 and MFI membranes, as well as those of traditional MMMs. The high-level and the proposed concept of C_3_H_6_/C_3_H_8_ separation redefined the upper bound and initiated a new generation of membranes for molecular separation applications. The characteristics and the intrinsic disadvantages of general MMMs and the nanoporous hybrid membranes are summarized in Table 8. The detailed separation properties of MMMs were summarized in Table 9.

**Figure 9:**
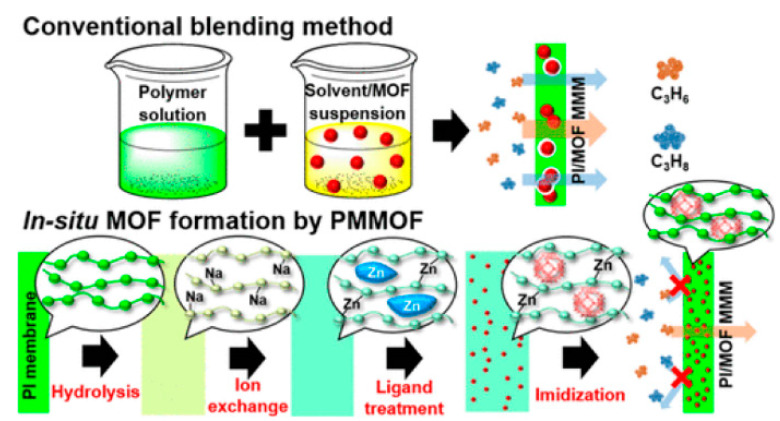
Schematic image of the in situ formation of metal organic framework (MOF)-doped MMMs [77].

Most of the reported MMMs are synthesized by mixing two individual phases (a dispersed phase and a continuous phase). However, the critical conditions must be controlled to suppress the possible generation of interfacial voids. Recently, the concept of in-situ growth of MOFs in a polymer matrix was proposed for the fabrication of MMMs for gas separation [78,79,80]. As revealed in Figure 9, four steps are required: polymer hydrolysis, ion exchange, ligand treatment, and imidization for the in situ formation of MMMs [77]. The in situ growth method potentially eliminates the formation of interfacial voids and enhances the dispersity of inorganic fillers in the polymer matrix. In addition, the resultant novel MMMs showed much higher C_3_H_6_/C_3_H_8_ selectivities than that of the conventional MMMs. The authors also demonstrated the possible scalability of the MMMs derived by the in situ growth method using commercial hollow fiber membranes, which also exhibited acceptable C_3_H_6_/C_3_H_8_ separation properties (C_3_H_6_ permeance: 2.17 GPU, C_3_H_6_/C_3_H_8_ selectivity: ~20).

## 3. Organosilica Membranes

Compared to pure silica membranes, organosilica membranes exhibit enhanced hydrothermal stability, lower permeation resistance, and wider applicability due to the incorporation of organic groups [81,82,83,84]. Since the first round of the successful application of organosilica membranes derived from bis(triethoxysilyl)ethane (BTESE) in 2008 [85], the research interest on silica membranes has changed and transitioned to the exploitation of novel organosilica membranes. BTESE membranes obtained from the conventional sol–gel process demonstrated excellent dehydration properties in separating *n*-butanol/water (95:5) mixtures [85]. The water concentration on the permeation side still remained at 98 wt% during on-stream operation for 1.5 years, showing great promise for practical applications. Our group proposed the concept of the “spacer technique” by the incorporation of bridged organics, which was confirmed to be effective for tailoring the network sizes of conventional silica membranes [36,86]. Subsequently, the number of studies on the gas separation [87,88,89,90,91,92,93], pervaporation [39,90,94], vapor permeation [40,95], and reverse osmosis [37,38,96] using organosilica membranes rapidly increased. Some of the organosilica membranes have also shown great prospects for C_3_H_6_/C_3_H_8_ separation, and considerable detailed work has been performed [45,89,93,97,98,99,100,101,102]. In this section, we will discuss the progress of C_3_H_6_/C_3_H_8_ separations using organosilica membranes. The synthesis approaches and the pore subnano-environment engineering of organosilica membranes are summarized in detail.

### 3.1. Synthesis of Organosilica Membranes

**Figure 10:**
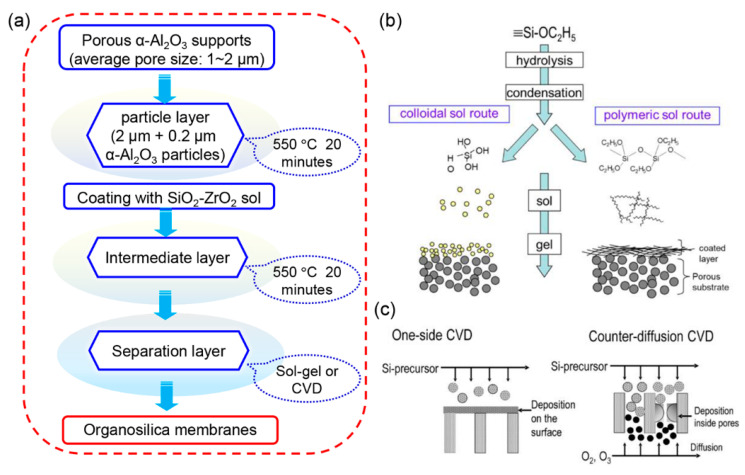
(**a**) Schematic illustration of the detailed fabrication process of organosilica membranes. (**b**) Sol–gel process for the formation of a separation layer including polymeric and colloidal sol routes [81]. (**c**) One-sided chemical vapor deposition (CVD) and counter-diffusion CVD methods for the deposition of an organosilica layer [81].

Generally, porous α-Al_2_O_3_ ceramics were used as the supports to fabricate organosilica membranes. Prior to the preparation of the top layer, the formation of particle layer and intermediate layer are necessary to reduce the pore size, as demonstrated in Figure 10a. Once the pore size of the intermediate layer is confirmed to be suitable for the deposition of the top layer, the sol–gel method [36,38,39,45,87,88,89,91,92,93,96,97,98,99,100,101,103] (Figure 10b) or the chemical vapor deposition (CVD) technique [104,105,106,107] (Figure 10c) can be conducted for the fabrication of an organosilica selective layer. In a typical sol–gel process, the polymeric and colloidal sol routes are divided based on the different reaction rates. The relatively lower reaction rate in the polymeric sol route allows partial hydrolysis. As a result, the formed organosilica layer is always a linear type with a micropore size lower than 1 nm. This process is commonly controlled using an acid as the catalyst or dropping a small amount of water for the hydrolysis reaction. In contrast, the utilization of a base as the catalyst and the dropping of excessive amount of water accelerate the reaction rate, generating a colloidal sol with abundant silanol (Si-OH) groups. Consequently, the molecules always permeate the interparticle pores within the membranes derived by the colloidal sol route. The overall time for the fabrication of organosilica membranes via sol-gel strategy is estimated to be around 4 h.

Another popular approach for the fabrication of organosilica membranes is the CVD technique, as presented in Figure 10c. The organosilica precursors flow to the surface of the support in a vapor phase, and subsequently, condensation reactions occur at temperature arounds 400–600 °C [107]. Compared to the membranes derived by the sol–gel method with higher permeances and moderate selectivities, the membranes fabricated from the CVD technique often exhibit dense structures with lower permeances but higher selectivities. Even though high-quality membranes can be obtained via the CVD technique, the organic groups have difficulty surviving at high temperatures. Hence, the plasma-enhanced CVD (PECVD) technique was extensively studied to replace the conventional CVD method, which allowed membrane fabrication at much lower temperatures [106,107,108,109]. Nagasawa et al. fabricated a series of organosilica membranes with different O/Si ratios via the PECVD technique [106]. The deposition time of the PECVD method was discussed, which revealed that stable gas permeation properties could be achieved within only 5 min. The PECVD technique effectively reduced the reaction temperature and the time consumption, which contributed to the easy fabrication of organosilica membranes. Nevertheless, no report of the scalable preparation of organosilica membranes used the PECVD strategy. The published papers related to PECVD-derived organosilica membranes are still limited in the lab-scale. In addition, the hollow fibers can be considered to act as the support instead of the ceramics to decrease the fabrication cost in the further studies.

**Figure 11:**
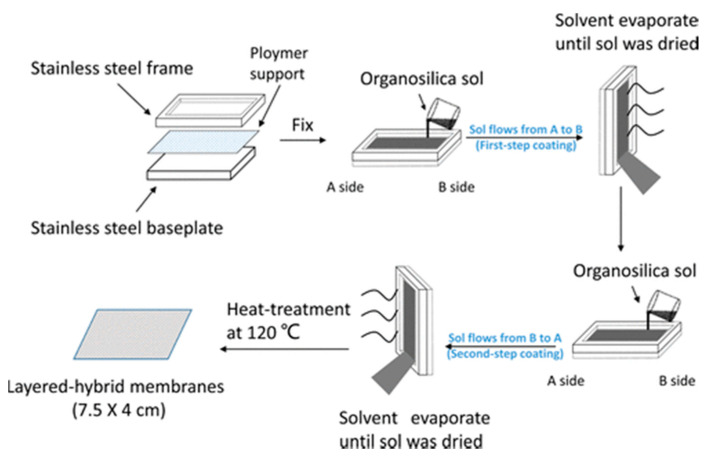
Schematic diagram for fabrication of organosilica/polymeric support membrane via the “flow-induced deposition” approach [111].

Most of the reported organosilica membranes, however, are fabricated on the expensive inorganic supports, which highly hampers the commercialization process. Therefore, the deposition of organosilica membranes on polymeric support is anticipated to be beneficial for achieving this target. Some successful cases have been reported by Gong et al., where the organosilica sols were spin-coated onto the surface of a porous polymeric support [40,110]. The resultant membranes showed stable dehydration performance for the water/isopropanol mixtures. Nevertheless, the spin-coating process is still not suitable for the industrial application, since it is difficult to be implemented on a large-area polymer membrane. To solve this problem, Gong et al. proposed an approach named “flow-induced deposition” for scalable fabrication of a continuous and uniform organosilica membrane on the porous polymeric support as shown in Figure 11 [111]. The “flow-induced deposition” derived organsilica membranes demonstrate high NaCl rejection in a reverse osmosis desalination process. Interestingly, this membrane still showed high stability and flexibility even rolled into a curvature radius of 11 mm. It is fact that the fabrication of organosilica membranes on polymeric support is feasible, however, the gas molecular sieving properties need to be significantly improved and the mechanical stability of polymeric support should also be carefully considered.

### 3.2. Pore Subnano-Environment Engineering of Organosilica Membranes

Generally, the pore subnano-environment engineering of organosilica membranes consists of pore size control and affinity control. Pore size control strategy includes spacer technique, incorporation of metal ions, and co-condensation reaction, respectively. In addition, affinity control method includes control of calcination parameters and post treatment.

#### 3.2.1. Pore Size Control

##### Spacer Technique

**Figure 12:**
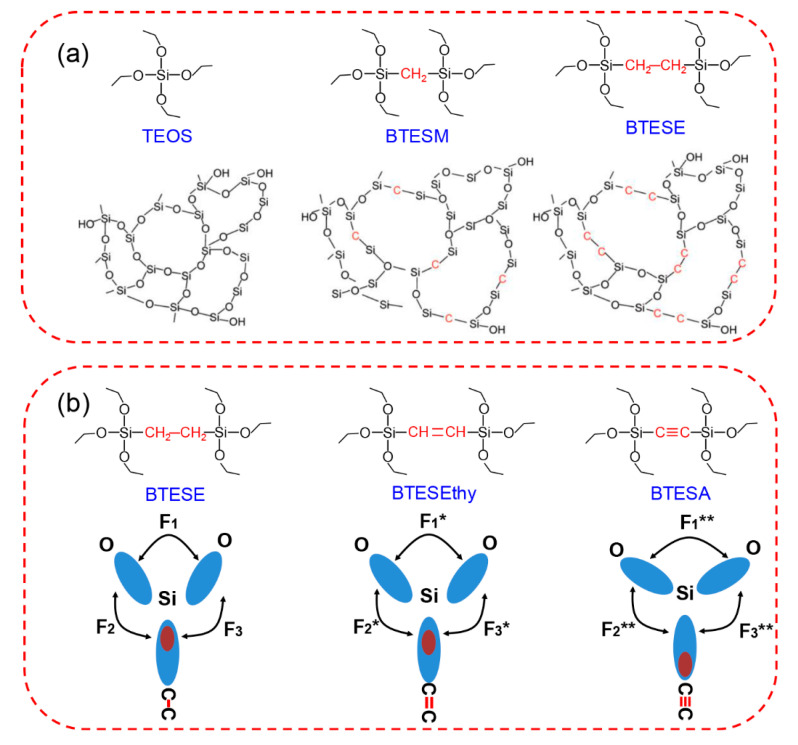
(**a**) Schematic illustration of amorphous network structures derived from tetraethoxysilane (TEOS), bis(triethoxysilyl)methane (BTESM), and bis(triethoxysilyl)ethane (BTESE) [89]. (**b**) Bonding structure model of BTESE-, BTESEthy-, and BTESA-derived networks [93].

The “spacer technique” proposed by our group is effective for the tuning of the membrane pore size, which is beneficial for various types of organosilica precursors [36,86,87,89,93,112]. In comparison with the pure silica membrane derived from tetraethoxysilane (TEOS), the existence of Si-R-Si units (R represents the organic bridge) in the organosilica networks changed the minimum constructive unit, and as a result, the network size can be efficiently adjusted. TEOS, bis(triethoxysilyl)methane (BTESM), and BTESE were selected as the precursors for the preparation of organosilica membranes, as shown in Figure 12a [89]. The single gas permeation properties clearly illustrated the effect of the precursors on the formation of amorphous structures. Based on the modified gas translation model, the average pore size of the BTESM membrane was determined to be between those of the TEOS and BTESE membranes. More importantly, BTESM membranes with bridges of the methane groups showed prospects for C_3_H_6_/C_3_H_8_ separation (C_3_H_6_ permeance of 6.32 × 10^−8^ mol m^−2^ s^−1^ Pa^−1^, C_3_H_6_/C_3_H_8_ selectivity around 8.8) due to the controlled network size. However, the C_3_H_6_/C_3_H_8_ separation performance of BTESM membranes still must be improved, including both the C_3_H_6_ permeance and C_3_H_6_/C_3_H_8_ selectivity.

Recently, we proposed a novel strategy to tailor the microstructures and permeation properties of organosilica membranes via control of the bond angles, as shown in Figure 12b [93]. Sol–gel derived organosilica membranes with different linking groups (ethane, ethylene, and acetylene) were fabricated using BTESE, bis(triethoxysilyl)ethylene (BTESEthy), and bis(triethoxysilyl)acetylene (BTESA) precursors. The microstructure and gas permeation properties were successfully tailored by controlling the Si-O-Si and Si-CC bond angles. The changes of the Si-CC bond angles were easily confirmed by the different conformations of the bridged groups of BTESE, BTESEthy, and BTESA, which were attributed to the different unsaturated degrees of the bridges. A nearly linear shape of the Si-C≡C bonds indicated the rigid structures of the BTESA membranes. In the case of the Si-O-Si bonds, the bonding structure model was utilized to interpret this interesting evolution trend. The locations of the electrons in the Si-C≡C bonds tended to be much further from Si atoms than those in Si-C-C and Si-C=C bonds, which enhanced the electron pair repulsion (F_1_** > F_1_* > F_1_). The increases in the Si-O-Si and Si-CC bond angles contributed to the construction of a loose and uniform structure, which was also evidenced by the FT-IR spectra. BTESA membranes featured a more open and accessible pore structure, which was found to be suitable for the separation of C_3_H_6_/C_3_H_8_. BTESA membranes with acetylene-bridged groups showed a superior C_3_H_6_ permeance of 1.0–2.0 × 10^−7^ mol m^−2^ s^−1^ Pa^−1^ (299–597 GPU) with an ideal C_3_H_6_/C_3_H_8_ selectivity of 11–14 in single gas permeation measurements, demonstrating great potential for membrane-based hydrocarbon separations.

The “spacer technique” has been extensively confirmed to be effective for the adjustment of the membrane network structure by varying the bridged groups. Various kinds of precursors have been utilized from the large organosilica family for the fabrication of membranes. Many exciting achievements have been made, and some of these membranes were successfully meet current industrial demands and expectations [113]. For the specific molecular separation process, however, an objective screening of the organosilica precursors should be carefully conducted.

##### Incorporation of Metal Ions

**Figure 13:**
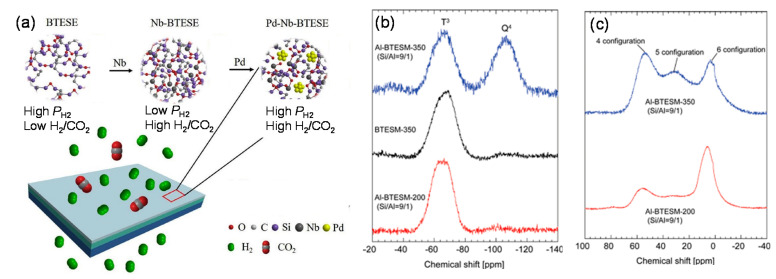
(**a**) Schematic image of the possible structures of Pd–Nb–BTESE networks for H_2_/CO_2_ separation [114]. (**b**) ^29^Si and (**c**) ^27^Al magic-angle spinning nuclear magnetic resonance (MAS NMR) spectra for BTESM and Al-BTESM gel powders [100].

The effectiveness of metal incorporation on the adjustment of the membrane pore structures has also been evidenced for organosilica membranes (Figure 13) [100,114]. As reported by Kanezashi et al., the sol–gel technique was applied to prepare Al-BTESM membranes for improved C_3_H_6_/C_3_H_8_ separation [100]. The ^29^Si and ^27^Al magic-angle spinning nuclear magnetic resonance (MAS NMR) spectra shown in Figure 13b,c confirmed the existence of Al in the composite network structures. A satisfyingly high C_3_H_6_/C_3_H_8_ selectivity of around 40 with a C_3_H_6_ permeance of 6.3 ✕ 10^−9^ mol m^−2^ s^−1^ Pa^−1^ (18.8 GPU) was achieved, which was attributed to the fine adjustment of the membrane pore size by the incorporation of Al in the BTESM-derived networks and/or through coordination with silanol (Si-OH) groups. Subsequently, the effect of the Al content on the network size of composite membranes was investigated [97]. The permeance of each gas molecule decreased, while the selectivity of H_2_/CH_4_ and H_2_/C_3_H_8_ increased as the Al content increased. The densified structure was also indicated by the increased activation energies of He, H_2_, N_2_, and CH_4_. However, although the C_3_H_6_/C_3_H_8_ selectivity was clearly enhanced by the introduction of Al, the significant loss of the C_3_H_6_ permeance cannot be ignored. For instance, the C_3_H_6_/C_3_H_8_ selectivity enhanced from 20 to 30 with an approximately one-order-of-magnitude decrease in the C_3_H_6_ permeance (from 6.5 ✕ 10^−8^ to 6.3 ✕ 10^−9^ mol m^−2^ s^−1^ Pa^−1^, 194 to 18.8 GPU) at 50 °C when BTESM and Al-BTESM (Si-Al 9:1) membranes were compared.

##### Co-Condensation Reaction

Organosilica membranes have exhibited potential for molecule separation. However, sometimes the individual organosilica-precursor-derived membranes cannot simultaneously achieve excellent molecular sieving properties, high hydrothermal stability, and specific interactions with the permeated molecules. Consequently, a co-condensation strategy was proposed to fabricate composite organosilica membranes by mixing different precursors. The membrane structure and the resultant separation performance can be finely controlled by varying the mixing ratios.

A BTESE-TEOS composite membrane with a controlled pore size was prepared via the co-hydrolysis and condensation method [115]. The single gas permeation properties and the estimated pore size determined by the normalized Knudsen-based permeance indicated that the network size of a composite membrane was well tuned between the individual BTESE- and TEOS-derived membranes. Additionally, the composite BTESE-TEOS membrane exhibited a superior O_2_ permeance which was higher than 10^−8^ mol m^−2^ s^−1^ Pa^−1^ with an O_2_/SO_2_ selectivity of 7.3, revealing the prospects for O_2_/SO_2_ separation of this composite membrane. Yu et al. tailored the microporosity properties of organosilica membranes by the co-condensation reaction of 4,6-bis(3-(triethoxysilyl)-1-propoxy)-1,3-pyrimidine with BTESE and/or TEOS [92]. A significantly enhanced CO_2_ permeance (>2000 GPU) with a CO_2_/N_2_ selectivity of ~20 was recorded for the composite organosilica membrane, showing potential for the highly permeable CO_2_ capture process.

**Figure 14:**
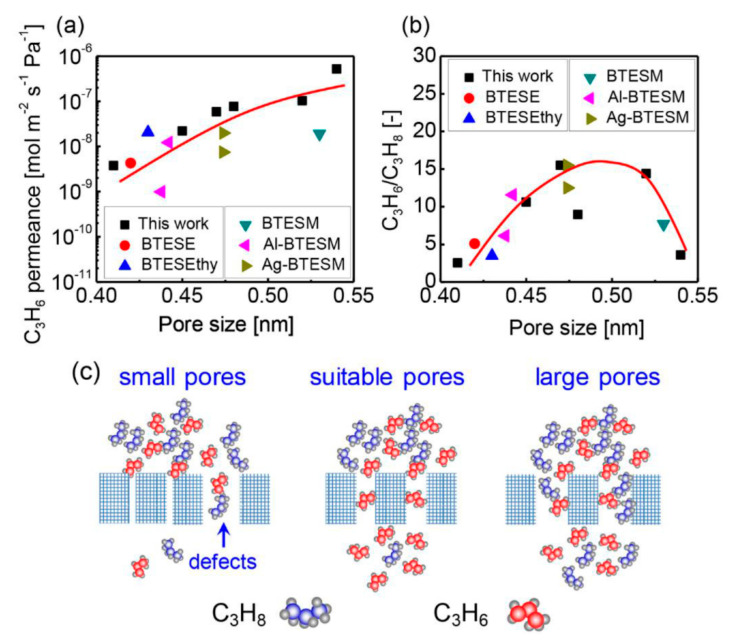
Relationships between pore size and single gas permeation properties: (**a**) C_3_H_6_ permeance and (**b**) C_3_H_6_/C_3_H_8_ permeance ratio at 200 °C for a variety of (composite) organosilica membranes. (**c**) Possible schematic image for C_3_H_6_/C_3_H_8_ separation utilizing organosilica membranes with different pore sizes [101].

Assisted by the rigid acetylene bridges, BTESA membranes featured a more open and accessible pore structure, showing prospects in C_3_H_6_/C_3_H_8_ separation [93]. To further advance the membrane separation performance, we fabricated a composite organosilica membrane using BTESA and bis(triethoxysilyl)benzene (BTESB) precursors [101]. The evolution of membrane networks revealed that the steric hindrance of phenyl groups originating from the BTESB played a key role in fine-tuning the membrane pore size. The BTESAB 9:1 (molar ratio of BTESA to BTESB was 9:1) membrane exhibited a high C_3_H_6_ permeance of 4.5 × 10^−8^ mol m^−2^ s^−1^ Pa^−1^ (134 GPU) together with a C_3_H_6_/C_3_H_8_ selectivity of 33 at 50 °C for an equimolar mixture of C_3_H_6_ and C_3_H_8_. More importantly, the relationships between the membrane pore size and C_3_H_6_/C_3_H_8_ separation performance for organosilica membranes in single and binary separation systems were clarified and summarized in detail. As depicted in Figure 14, we investigated the relationship between the membrane pore size (as determined by the modified gas translation model) and the membrane separation performance including the C_3_H_6_ permeance and C_3_H_6_/C_3_H_8_ selectivity. The adopted data were measured at 200 °C to minimize the adsorption effect between the hydrocarbon molecules and membrane materials. In other words, the molecular sieving effect dominated the separation process at this high temperature.

Membranes with pores that are too small cannot provide enough channels for gas permeation. As a result, C_3_H_6_ and C_3_H_8_ only permeate through the defects with low selectivity properties. In contrast, membranes with sufficiently large pores can drastically increase the gas permeance. However, these large pores allow both C_3_H_6_ and C_3_H_8_ molecules to easily permeate without any major resistance or effective molecular sieving properties. In contrast to membranes with pores that are too small or too large, membranes with suitable pore sizes feature much higher selectivities due to the good molecular sieving properties. The suitable pore size determined by modified a gas translation (GT) model ranged from 0.45 to 0.52 nm, which fell approximately between the reported molecular size of C_3_H_6_ (0.468 nm) and C_3_H_8_ (0.506 nm), providing the possibility to discriminate and separate C_3_H_6_ and C_3_H_8_ molecules [101]. This explanation can quantify the relationships between the membrane pore sizes and C_3_H_6_/C_3_H_8_ separation properties and explain why membranes with pores that are too large or too small pores are not suitable for high-efficiency separations.

#### 3.2.2. Affinity Control

##### Calcination Parameters

Calcination treatment is necessary to facilitate the formation of organosilica networks in a typical sol–gel process. Hence, the control of calcination parameters, such as the temperature, heating/cooling rate, and calcination atmosphere, is particularly important for manufacturing high-quality membranes [39,45,98,116,117].

BTESM membranes calcined at 200 °C, 350 °C, and 600 °C were reported by Kanezashi et al. to illustrate the different gas permeation behaviors [98]. The ratio of the C_3_H_6_/C_3_H_8_ selectivities obtained from a binary separation system and a single gas permeation system for the BTESM membrane calcined at 200 °C (BTESM-200) was higher than that of BTESM membrane calcined at 350 °C (BTESM-350). The permeation of C_3_H_6_ molecules might have shown higher efficiency in blocking the permeation of C_3_H_8_ molecules for BTESM-200 than BTESM-350, because of the higher density of Si-OH groups in BTESM-200 can interact with the C_3_H_6_ molecules. In addition, it should be noted that the combustion of bridged -CH_2_- bonds and the construction of Si-O-Si groups simultaneously occurred when a BTESM membrane was calcined at 600 °C, leading to the formation of a silica-like network with a densified structure.

**Figure 15:**
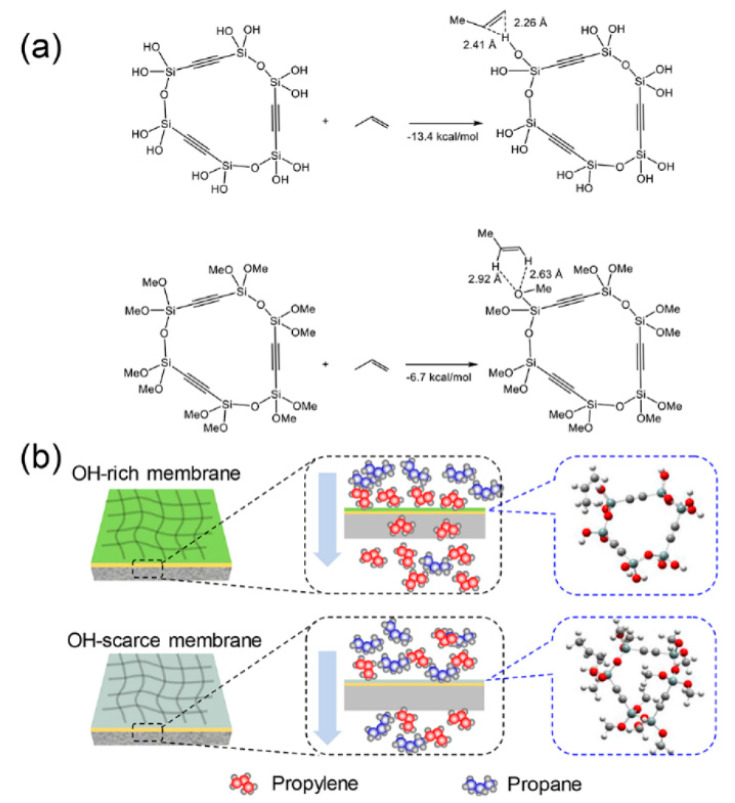
(**a**) Model reactions cyclic trimers and optimized geometries of propylene complexes. (**b**) Plausible C_3_H_6_/C_3_H_8_ separation mechanisms through BTESA membranes with different chemical properties [45].

Recently, the C_3_H_6_/C_3_H_8_ separation performances of BTESA membranes fabricated at different temperatures were also extensively studied [45]. FT-IR, surface energy, and hydrocarbon sorption isotherm characterization revealed that low-temperature calcined BTESA materials with more Si-OH groups exhibited enhanced affinities for C_3_H_6_ molecules. A molecular simulation (Figure 15a) also indicated that the hydrogen bonding between the Si-OH and C_3_H_6_ π-bonds enhanced the C_3_H_6_ affinity of the membrane. BTESA membranes calcined at 150 °C featured a high C_3_H_6_/C_3_H_8_ selectivity of 52 and C_3_H_6_ permeance of 1.7 × 10^−8^ mol m^−2^ s^−1^ Pa^−1^ (51 GPU) at 50 °C. Figure 15b presents the plausible C_3_H_6_/C_3_H_8_ separation mechanisms of BTESA membranes with different chemical properties. In contrast to an OH-scarce membrane, an OH-rich membrane accelerated the permeation of C_3_H_6_ molecules via stronger interactions and enhanced the C_3_H_6_/C_3_H_8_ separation properties simultaneously. Interestingly, the network sizes of BTESA membranes calcined at higher temperatures tended to be enlarged, which was opposite to the phenomenon found in BTESM membranes. The decomposition of rigid acetylene bridges resulted in a greater porosity rather than densifying the membrane structures.

##### Post Treatment

The Si-OH groups within the membrane network structures can accelerate the preferential permeation of C_3_H_6_ molecules and enhance the C_3_H_6_/C_3_H_8_ separation performance, which has been evidenced by BTESM- and BTESA-derived membranes [45,98]. Consequently, determining how to effectively increase the Si-OH density is significant for advancing the C_3_H_6_/C_3_H_8_ separation performance. Kanezashi et al. prepared a microporous silica membrane utilizing triethoxyfluorosilane (TEFS) as the Si source [118]. It should be emphasized that TEFS is not an organosilica precursor in the strict sense, since no organic groups can be observed. Nevertheless, the similar amorphous structure and the gas permeation mechanism of TEFS and organosilica membranes may still encourage the exploitation of membranes with high permselectivities.

**Figure 16:**
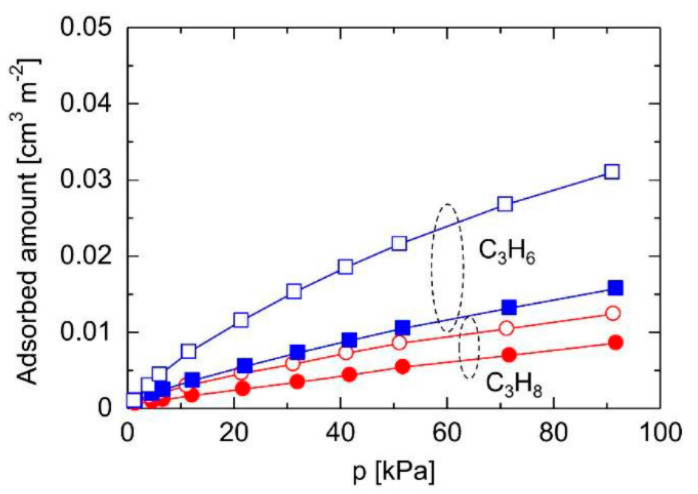
C_3_H_6_ and C_3_H_8_ adsorption isotherms at 25 °C for the triethoxyfluorosilane (TEFS) powders calcined at 350 °C before/after steam treatment (closed symbols: before steam treatment, open symbols: after steam treatment) [118].

A steam treatment strategy was proposed to increase the Si-OH densities of TEFS membranes via the reaction between steam and Si-F bonds. As revealed in Figure 16, the gas sorption isotherms demonstrated that the adsorbed amount of C_3_H_6_ was significantly increased for TEFS powders with steam treatment, while that of C_3_H_8_ was almost the same as that of the pristine TEFS powder. In a single gas permeation system, the TEFS membrane with steam treatment featured a superior C_3_H_6_ permeance of 2.2 ✕ 10^−7^ mol m^−2^ s^−1^ Pa^−1^ (657 GPU) with a C_3_H_6_/C_3_H_8_ selectivity of 42. However, the binary separation properties were not determined for this membrane. Inspired by this, the strategy of steam treatment may be applied for BTESM and BTESA membranes. Additionally, H_2_O/N_2_ plasma modification technology can also be implemented to achieve a high grafting efficiency of OH groups while causing no damage to the surface morphology and bulk chemistry [119]. The specific performances of the reported (organo)silica membranes are provided in Table 10.

## 4. Conclusions and Outlook

**Figure 17:**
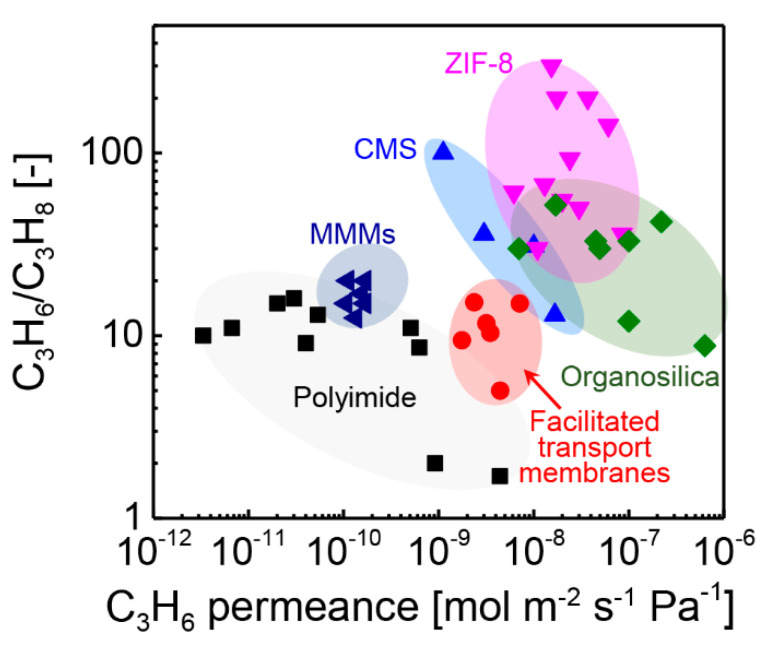
Trade–off of C_3_H_6_/C_3_H_8_ for polyimide [20,46], MMMs [27,120], facilitated transport membranes [22,23], CMS [31,33,52,53], ZIF–8 [17,65,66,67,68,69,72,73,74,75,76], and organosilica [45,89,93,97,98,99,101,118] membranes.

In this article, we surveyed the progress of membrane-based separation processes of C_3_H_6_/C_3_H_8_. In Figure 17, the trade-off for the C_3_H_6_/C_3_H_8_ separation of several kinds of membranes is provided. The specific values of each membranes are summarized in Table S1. The polymeric membranes and MMMs are located at the left-bottom of the trade-off, indicating relatively lower C_3_H_6_ permeance and C_3_H_6_/C_3_H_8_ selectivity values. In comparison, the inorganic membranes, including CMS, ZIF-8, and organosilica, are located at the opposite position. The inorganic membranes exhibited much higher permeance and selectivity values. However, the cost of the membrane materials should not be ignored.

Separation mechanisms, including solution-diffusion, facilitated transport, and molecular sieving, dominate the discrimination of C_3_H_6_ and C_3_H_8_ molecules through membranes. The separation performances of organic, inorganic, and hybrid membranes were highlighted, although they are still hampered by some inherent drawbacks. In addition, we summarized the recent developments of organosilica membranes in detail for the application of C_3_H_6_/C_3_H_8_ separation. The sol–gel method and the CVD strategy are usually used to fabricate the organosilica membranes. To advance the C_3_H_6_/C_3_H_8_ separation properties, pore subnano-environment engineering consisting of pore size control (spacer technique, incorporation of metal, and co-condensation strategy) and affinity control (calcination parameters and post treatment) are conducted on silica or organosilica membranes. BTESM and BTESA membrane materials have been shown to be suitable and exhibit great potential for C_3_H_6_/C_3_H_8_ separation, which is challenging because of the similar physico-chemical properties of C_3_H_6_ and C_3_H_8_ molecules.

However, some aspects must be examined in future studies of C_3_H_6_/C_3_H_8_ separation using organosilica membranes. First, no successful industrial application of organosilica membranes for C_3_H_6_/C_3_H_8_ separation has been achieved. New manufacturing strategies for scalable organosilica membranes must be further exploited for the practical separation of C_3_H_6_ and C_3_H_8_ mixtures. Low-cost supports such as hollow fibers can also be applied.

Second, the amorphous structures of organosilica membranes always generate a network with a pore size distribution rather than a network with a uniform pore size. As a result, the utilization efficiency of useful pores for C_3_H_6_/C_3_H_8_ separation is limited to a relatively low level. The template method using a surfactant contributes to the formation of uniform pores of organosilica materials, which is commonly known as periodic mesoporous organosilica (PMO). However, the pore sizes of these material are intrinsically too large for C_3_H_6_/C_3_H_8_ separation. Hence, the effective decrease in the pore size derived from template methods to the molecular scale is the key to obtaining novel organosilica membranes with superior C_3_H_6_ permeances and C_3_H_6_/C_3_H_8_ selectivities simultaneously. Grafting modification using a pendant-type organosilica precursor with long chains may be an alternative candidate.

Finally, the assessment of the affinity between C_3_H_6_ or C_3_H_8_ molecules and organosilica membranes is currently conducted through the qualitatively measurement of the single-component adsorption isotherms, in which conditions deviate significantly from the real conditions of binary separations of C_3_H_6_/C_3_H_8_ mixtures. The binary gas sorption properties or a measurement of the breakthrough may provide more useful information to better understand the separation mechanism of C_3_H_6_/C_3_H_8_ through organosilica membranes.

## Figures and Tables

**Table 1:** Summary of physical properties of C_3_H_6_ and C_3_H_8_ molecules.

Molecule	Kinetic Diameter [nm]	L-J Constant [nm]	Polarizability [10^25^ cm^3^]	Molecular Weight [g mol^−1^]	Boiling Temperature [°C]
C_3_H_6_	0.45	0.468	62.6	42.08	−42.1
C_3_H_8_	0.43	0.506	62.9~63.7	44.10	−47.6

**Table 2:** Separation performance of C_3_H_6_/C_3_H_8_ for polyimide membranes.

Membrane	Thickness [μm]	Preparation Method	Feed Propylene Pressure	C_3_H_6_ Permeance [barrer]	C_3_H_6_/C_3_H_8_ Selectivity [-]	Temperature [°C]	Ref.
6FDA	20~30	Casting	0.38	0.38	13	35	[20]
6FDA	20~30	Casting	1.30	0.48	12.8	35	[20]
6FDA	20~30	Casting	1.85	0.66	8	35	[20]
6FDA	20~30	Casting	2.02	0.68	7.2	35	[20]
6FDA ^a^	25.4	Casting	-	0.85	17	35	[19]
KAUST-PI-1 ^a^	100	Casting	-	817	16	35	[47]

^a^ Ideal gas separation performance; C_3_H_6/_C_3_H_8_ mixtures composition (5/5).

**Table 3:** Separation performance of C_3_H_6_/C_3_H_8_ for facilitated transport membranes.

Membrane	Thickness [μm]	C_3_H_6_ Permeability [barrer]	C_3_H_6_/C_3_H_8_ Selectivity [-]	Temperature [°C]	Ref.
cPIM	70~90	421	9.42	35	[22]
AgcPIM	70~90	759	11.69	35	[22]
MgcPIM	70~90	568	15.23	35	[22]
ZncPIM	70~90	837	9.12	35	[22]
Ag-ion gel membrane	37.5	850	15	25	[23]

**Table 4:** Electronegativity of the transition metals [5].

Electronegativity of the Transition Metals								
**Sc**	**Ti**	**V**	**Cr**	**Mn**	**Fe**	**Co**	**Ni**	**Cu**
1.4	1.5	1.6	1.7	1.6	1.8	1.9	1.9	1.9
**Y**	**Zr**	**Nb**	**Mo**	**Tc**	**Ru**	**Rh**	**Pd**	**Ag**
1.3	1.3	1.6	2.2	1.9	2.2	2.3	2.2	2.2
**La**	**Hf**	**Th**	**W**	**Re**	**Os**	**Ir**	**Pt**	**Au**
1.0	1.3	1.5	2.4	1.9	2.2	2.2	2.3	2.5

**Table 5:** Characteristics and intrinsic drawbacks of inorganic membranes used for C_3_H_6_/C_3_H_8_ separation.

Membrane Types	Characteristics	Disadvantages
CMS	CMS membranes are fabricated from the pyrolysis of microporous polymers. Good processability and molecular sieving properties [49].	Fragile [50].
Zeolite	Zeolite membranes are widely used based on the controllable pore size and strong adsorption properties [13].	Grain boundary defects. Bad synthesis reproducibility [35].
ZIFs	The zeolite-like structure endows the ZIFs materials with high thermal and chemical stabilities [15].	High cost of membrane fabrication. Low reproducibility and scalability [15].

**Table 6:** Separation performance of C_3_H_6_/C_3_H_8_ for CMS membranes.

Membrane	Thickness [μm]	Preparation Method	C_3_H_6_ Permeance [10^−10^ mol m^−2^ s^−1^ Pa^−1^]	C_3_H_6_/C_3_H_8_ Selectivity [-]	Temperature [°C]	Ref.
6FDA	1.6	Dip-coating, pyrolysis	30	36	25	[52]
6FDA	0.52	Dip-coating, pyrolysis	100	31	~50	[33]
BPDA-DDBT	0.2	Dip-coating, pyrolysis	167	13	100	[53]
6FDA/BPDA-DAM	35~60	Casting, pyrolysis	11.2	100	35	[31]

**Table 7:** Separation performances of C_3_H_6_/C_3_H_8_ for zeolitic imidazolate framework (ZIF) membranes.

Membrane	Thickness [μm]	Preparation Method	Measurement Method	C_3_H_6_ Permeance [10^−10^ mol m^−2^ s^−1^ Pa^−1^]	C_3_H_6_/C_3_H_8_ [-]	Temperature [°C]	Ref.
ZIF-8	2.2	Seeded growth	W-K technique	300	50	−15	[65]
ZIF-8	2.5	Secondary growth	W-K technique	110	30	35	[72]
ZIF-8	1.5	Counter diffusion	W-K technique	200	55	25	[17]
ZIF-8 ^a^	1	seeding-free aqueous synthesis	-	850	36	25	[73]
ZIF-8	0.5	aqueously cathodic deposition	W-K technique	610	142	22	[69]
ZIF-8	2.5	hydrothermal synthesis	W-K technique	62	61	22	[66]
ZIF-8	-	secondary growth	W-K technique	240	93	35	[74]
ZIF-8	2	secondary growth	W-K technique	130	67	35	[75]
ZIF-8	0.2	Fast current–driven synthesis	-	154	300	25	[67]
ZIF-67	0.7	heteroepitaxially growing	W-K technique	370	200	25	[76]
ZIF-67	0.7	Fast current–driven synthesis	-	175	200	25	[68]

^a^ Ideal gas separation performance.

**Table 8:** Characteristics of mixed matrix membranes (MMMs) used for C_3_H_6_/C_3_H_8_ separation.

Membrane Types	Characteristics	Disadvantages
General MMMs	The general MMMs are fabricated by combining molecular sieve materials with traditional polymers, obtaining the advantages from both the high porosity of molecular sieves and good processability of the polymers [13].	The compatible issues between the inorganic filler and the polymer matrix. The permeability of membranes is still constrained by the matrix.
All nanoporous hybrid membranes	Both the filler and matrix are the nanoporous materials [30].	No report at the present.

**Table 9:** Separation performance of C_3_H_6_/C_3_H_8_ for MMMs.

Membrane	Thickness [μm]	C_3_H_6_ Permeance [GPU]	C_3_H_6_/C_3_H_8_ Selectivity [-]	Ref.
Zr-fum-fcu-MOF/6FDA-DAM	70	0.29~0.43	15~20	[27]
ZIF-8/6FDA-DAM	-	0.27	27.5	[28]
ZIF-8/PIM-6FDA-OH	40~60	0.5~0.75	31	[29]
ZIF-8/MFI	9 ± 3	61~68	146 ± 14	[30]
ZIF-8/PIM-6FDA-OH	0.75	2.17	20	[77]

**Table 10:** C_3_H_6_/C_3_H_8_ separation performances of (organo)silica membranes.

Membrane	Preparation Method	C_3_H_6_ Permeance [10^−10^ mol m^−2^ s^−1^ Pa^−1^]	C_3_H_6_ Permeance [GPU]	C_3_H_6_/C_3_H_8_ Selectivity [-]	Temperature [°C]	Ref.
BTESM ^a^	Sol-gel, wipe coating	6320	1888	8.8	50	[89]
BTESM	Sol-gel, wipe coating	495	142	30	50	[98]
Al-BTESM ^a^	Sol-gel, wipe coating	70	20	30	50	[97]
Ag-BTESM ^a^	Sol-gel, wipe coating	1000	287	33	50	[99]
BTESA ^a^	Sol-gel, wipe coating	1000	287	12	50	[93]
BTESA-B	Sol-gel, wipe coating	450	134	33	50	[101]
BTESA	Sol-gel, wipe coating	170	51	52	50	[45]
TEFS ^a^	Sol-gel, wipe coating	2200	657	42	35	[118]

^a^ Ideal gas separation performance.

## Supplementary Materials

The following are available online at https://www.mdpi.com/article/10.3390/membranes11050310/s1, Table S1: The detailed separation performance of various kinds of membranes.
