# Supplementary material for: Recent Progress in a Membrane-Based Technique for Propylene/Propane Separation
Source: Membranes (Basel). 2021 Apr 23;11(5):310. doi: 10.3390/membranes11050310 (PMC8145504; doi:10.3390/membranes11050310)
Supplement: Supplementary file 1 [file membranes-11-00310-s001.zip › membranes-1136181-supplementary.pdf]

Supporting Information

Table S1 The detailed separation performance of various kinds of membranes

Membrane	C_3H_6 permeance [mol m^{-2} s^{-1} Pa^{-1}]	C_3H_6/C_3H_8 selectivity [-]	Ref.
Polymer	2.01~3.35×10^{-12}	10~16*	1
	6.7×10^{-12}~4.4×10^{-9}	1.7~13	2
Facilitated transport membranes	1.7~3.5×10^{-9}	9.4 ~ 15	3
	4.5~7.1×10^{-9}	5 ~ 15	4
CMS	3×10^{-9}	36	5
	1×10^{-8}	31	6
	1.67×10^{-8}	13	7
	1.12×10^{-9}	100	8
ZIF	3×10^{-8}	50	9
	1.1×10^{-8}	30	10
	2×10^{-8}	55	11
	8.5×10^{-8}	36	12
	6.1×10^{-8}	142	13
	6.2×10^{-9}	61	14
	2.4×10^{-8}	93	15
	1.3×10^{-8}	67	16
	1.54×10^{-8}	300	17
	3.7×10^{-8}	200	18
	1.75×10^{-8}	200	19
Organosilica	6.32×10^{-7}	8.8	20
	4.95×10^{-8}	30	21
	7×10^{-9}	30	22
	1×10^{-7}	33	23
	1×10^{-7}	12	24
	4.5×10^{-8}	33	25
	1.7×10^{-8}	52	26
	2.2×10^{-7}	42	27
MMMs	1.05~1.68×10^{-10}	15~20	28
	1.34~1.68×10^{-10}	12.5~15	29

References

[1] C. Staudt-Bickel, W.J. Koros, Olefin/paraffin gas separations with 6FDA-based polyimide membranes, *J. Membr. Sci.* 170 (2000) 205-214.

[2] K. Tanaka, A. Taguchi, J. Hao, H. Kita, K. Okamoto, Permeation and separation properties of polyimide membranes to olefins and paraffins, *J. Membr. Sci.* 121 (1996) 197-207.

[3] K.-S. Liao, J.-Y. Lai, T.-S. Chung, Metal ion modified PIM-1 and its application for propylene/propane separation, *J. Membr. Sci.* 515 (2016) 36-44.

[4] S. Kasahara, E. Kamio, R. Minami, H. Matsuyama, A facilitated transport ion-gel membrane for propylene/propane separation using silver ion as a carrier, *J. Membr. Sci.* 431 (2013) 121-130.

[5] X. Ma, B.K. Lin, X. Wei, J. Kniep, Y. Lin, Gamma-alumina supported carbon molecular sieve membrane for propylene/propane separation, *Ind. Eng. Chem. Res.* 52 (2013) 4297-4305.

[6] X. Ma, Y. Lin, X. Wei, J. Kniep, Ultrathin carbon molecular sieve membrane for propylene/propane separation, *AIChE J. 62* (2016) 491-499.

[7] Okamoto, K.-i.; Kawamura, S.; Yoshino, M.; Kita, H.; Hirayama, Y.; Tanihara, N.; Kusuki, Y., Olefin/paraffin separation through carbonized membranes derived from an asymmetric polyimide hollow fiber membrane. *Ind. Eng. Chem. Res.* **1999,** 38, (11), 4424-4432.

[8] Steel, K. M.; Koros, W. J., An investigation of the effects of pyrolysis parameters on gas separation proper-ties of carbon materials. Carbon 2005, 43, (9), 1843-1856.

[9] Pan, Y.; Li, T.; Lestari, G.; Lai, Z., Effective separation of propylene/propane binary mixtures by ZIF-8 membranes. *J. Membr. Sci.* 2012, 390, 93-98.

[10] Liu, D.; Ma, X.; Xi, H.; Lin, Y., Gas transport properties and propylene/propane separation characteristics of ZIF-8 membranes. *J. Membr. Sci.* 2014, 451, 85-93.

[11] Kwon, H. T.; Jeong, H.-K., In situ synthesis of thin zeolitic–imidazolate framework ZIF-8 membranes exhibiting exceptionally high propylene/propane separation. *J. Am. Chem. Soc.* 2013, 135, (29), 10763-10768.

[12] Tanaka, S.; Okubo, K.; Kida, K.; Sugita, M.; Takewaki, T., Grain size control of ZIF-8 membranes by seeding-free aqueous synthesis and their performances in propylene/propane separation. *J. Membr. Sci.* 2017, 544, 306-311.

[13] Wei, R.; Chi, H. Y.; Li, X.; Lu, D.; Wan, Y.; Yang, C. W.; Lai, Z., Aqueously Cathodic Deposition of ZIF‐8 Membranes for Superior Propylene/Propane Separation. *Adv. Funct. Mater.* 2019, 1907089.

[14] Yu, J.; Pan, Y.; Wang, C.; Lai, Z., ZIF-8 membranes with improved reproducibility fabricated from sputter-coated ZnO/alumina supports. *Chem. Eng. Sci.* 2016, 141, 119-124.

[15] Sheng, L.; Wang, C.; Yang, F.; Xiang, L.; Huang, X.; Yu, J.; Zhang, L.; Pan, Y.; Li, Y., Enhanced C_3H_6/C_3H_8 separation performance on MOF membranes through blocking defects and hindering framework flexibility by silicone rubber coating. *Chem. Commun.* 2017, 53, (55), 7760-7763.

[16] Li, J.; Lian, H.; Wei, K.; Song, E.; Pan, Y.; Xing, W., Synthesis of tubular ZIF-8 membranes for propylene/propane separation under high-pressure. *J. Membr. Sci.* **2020,** 595, 117503.

[17] Zhou, S.; Wei, Y.; Li, L.; Duan, Y.; Hou, Q.; Zhang, L.; Ding, L.-X.; Xue, J.; Wang, H.; Caro, J., Paralyzed membrane: Current-driven synthesis of a metal-organic framework with sharpened propene/propane separation. *Sci. Adv.* 2018, 4, (10), eaau1393.

[18] Kwon, H. T.; Jeong, H.-K.; Lee, A. S.; An, H. S.; Lee, J. S., Heteroepitaxially grown zeolitic imidazolate framework membranes with unprecedented propylene/propane separation performances. *J. Am. Chem. Soc.* 2015, 137, (38), 12304-12311.

[19] Hou, Q.; Zhou, S.; Wei, Y.; Caro, J.; Wang, H., Balancing the grain boundary structure and the framework flexibility through bimetallic MOF membranes for gas separation. *J. Am. Chem. Soc.* 2020, 142, (21), 9582–9586.

[20] Kanezashi, M.; Kawano, M.; Yoshioka, T.; Tsuru, T., Organic–inorganic hybrid silica membranes with con-trolled silica network size for propylene/propane separation. *Ind. Eng. Chem. Res.* 2011, 51, (2), 944-953.

[21] Kanezashi, M.; Shazwani, W.; Yoshioka, T.; Tsuru, T., Separation of propylene/propane binary mixtures by bis (triethoxysilyl) methane (BTESM)-derived

silica membranes fabricated at different calcination tempera-tures. *J. Membr. Sci.* 2012, 415, 478-485.

[22] Kanezashi, M.; Miyauchi, S.; Nagasawa, H.; Yoshioka, T.; Tsuru, T., Gas permeation properties through Al-doped organosilica membranes with controlled network size. *J. Membr. Sci.* 2014, 466, 246-252.

[23] Kanezashi, M.; Miyauchi, S.; Hayakawa, S.; Nagasawa, H.; Yoshioka, T.; Tsuru, T., Propylene/propane per-meation properties of metal-doped organosilica membranes with controlled network sizes and adsorptive properties. *J. Jpn. Pet. Inst.* 2016, 59, 140-148.

[24] Guo, M.; Kanezashi, M.; Nagasawa, H.; Yu, L.; Yamamoto, K.; Gunji, T.; Ohshita, J.; Tsuru, T., Tailoring the microstructure and permeation properties of bridged organosilica membranes via control of the bond an-gles. *J. Membr. Sci.* 2019, 584, 56-65.

[25] Guo, M.; Kanezashi, M.; Nagasawa, H.; Yu, L.; Yamamoto, K.; Gunji, T.; Tsuru, T., Fine‐tuned, molecu-lar‐composite, organosilica membranes for highly efficient propylene/propane separation via suitable pore size. *AlChE J.* 2020, 66, (4), e16850.

[26] Guo, M.; Kanezashi, M.; Nagasawa, H.; Yu, L.; Yamamoto, K.; Gunji, T.; Ohshita, J.; Tsuru, T., Pore sub-nano-environment engineering of organosilica membranes for highly selective propylene/propane separation. *J. Membr. Sci.* 2020, 117999.

[27] Kanezashi, M.; Matsutani, T.; Nagasawa, H.; Tsuru, T., Fluorine-induced microporous silica membranes: Dramatic improvement in hydrothermal stability and pore size controllability for highly permeable propyl-ene/propane separation. *J. Membr. Sci.* 2018, 549, 111-119.

[28] Liu, Y.; Chen, Z.; Liu, G.; Belmabkhout, Y.; Adil, K.; Eddaoudi, M.; Koros, W., Conformation-controlled molecular sieving eEffects for membrane-based propylene/propane separation. *Adv. Mater.* 2019, 31, (14), 1807513.

[29] Yu, J.; Wang, C.; Xiang, L.; Xu, Y.; Pan, Y., Enhanced C_3H_6/C_3H_8 separation performance in poly (vinyl acetate) membrane blended with ZIF-8 nanocrystals. *Chem. Eng. Sci.* 2018, 179, 1-12.
